# Typed Operation Notes in Rural Western Australia: Improving Patient Care

**DOI:** 10.7759/cureus.23407

**Published:** 2022-03-22

**Authors:** Amelia Davis, Dean Mckittrick, Nils Wagner

**Affiliations:** 1 General Surgery Department, Broome Hospital, Broome, AUS; 2 General Practice, Western Australia Country Health Service - Kimberley, Broome, AUS

**Keywords:** post-operative care, healthcare, operation report, general surgery, handwritten, typed, operation note

## Abstract

Introduction: Given the geographical area of the Kimberley region in Western Australia (WA) and the nomadic nature of its residents and medical staff, widespread access and clarity of surgical clinical information are necessary to provide accurate and timely post-surgical care. The aims of this project were: to evaluate the quality of operation notes and secondly, to evaluate multidisciplinary staff perceptions of the impact of the introduction of typed operation notes for general surgery in the Kimberley region from 2019 to 2020.

Methods: The quality of 100 general surgery operation notes (50 typed and 50 handwritten) were reviewed against the Royal College of Surgeons England (RCSEng) operation note guidelines. Cases were selected at random and reviewed by a resident medical officer. Multidisciplinary staff perceptions of communication were assessed through an anonymous electronic survey across emergency departments, general practices, nursing staff, and allied health staff members from the top three population centres: Broome, Derby and Kununurra.

Results: Typed operation notes with pre-loaded data (date, time, etc.) and mandatory fields (surgical count correct, etc.) increased recorded information and quality of content by 45% when compared to handwritten notes. When compared to RCSEng standards for free text, anticipated blood loss (one typed note) and abbreviation use (44 typed and 37 handwritten) showed ongoing user-dependent areas for improvement.

A review of multidisciplinary staff perceptions (79 returned surveys) showed 60% of handwritten notes were seen to have a negative impact on timely post-operative care. Overall, typed notes increased legibility with a perceived improvement in acronyms/abbreviations and completeness of documentation. More than 90% of staff suggested an extension of typed notes for all surgical operations would be beneficial.

Conclusion: The Kimberley region poses a unique set of challenges to the continuity of post-surgical care. This review shows typed operation notes improve legibility (100%) and increased congruence with established guidelines by 45%. It also shows a successful model for increased local and metropolitan multidisciplinary access across remote WA for timely post-operative care. In an unprecedented time where elective surgical procedures are being reduced to meet pandemic demands, now is the time to review, consider and institute practices that improve intra-operative and post-operative care.

## Introduction

The Kimberley is the northern-most region of Western Australia (WA) comprising 424,517 square kilometres with an estimated residential population of 36,230. There are over 100 Aboriginal communities, comprising approximately a third of the population with over 40 known dialects. The top three major population centres with hospitals within the Kimberley region are Broome, Kununurra, and Derby [1]. The vast geographical area, fluctuations in population size between seasons, and nomadic nature of its residents (both Indigenous and non-Indigenous Australians) and medical staff generate unique challenges to optimal post-operative care.

Surgical operation notes are the formal record of events in the operating room and are used as a communication tool between an array of multidisciplinary health team members perioperatively. As such, accurate and accessible documentation is paramount to patient safety and care. In accordance with the Australian Medical Association and the National Safety and Quality Health Service, good medical practice involves clear, effective, and prompt communication. It also requires the use of sufficient information to enable continued care by other doctors and multidisciplinary professionals [2,3]. In keeping with the Good Medical Practice (the Code), The Royal College of Surgeons of England (RCSEng) has clear guidelines for operation note requirements [4].

Handwritten notes in health care can have a reputation for being difficult to interpret and access. The effect of poor intra-operative documentation (operation notes) is known to impact negatively on patient safety, for example, incorrect surgical counts leading to unintended retention of foreign objects [5-7].

Medical documentation in the Kimberley region is largely paper-based, and operation notes are handwritten using a template. Subsequently, the amount of detail, adequacy, and legibility of information is reliant on the author of the document and is only available at the primary operative hospital. Accessing handwritten operation notes between hospitals (metropolitan and regional) involves contacting medical records during working hours (08:00-16:00) and awaiting a facsimile, which can take hours or days.

In 2019, a hybrid system was introduced into the Kimberley region with the introduction of typed operation notes for cases during working hours, and handwritten operation notes for after-hours (16:00-08:00) cases. This study aims to review the quality of general surgery typed operation notes and perceptions of multidisciplinary communication throughout the Kimberley.

## Materials and methods

General surgery typed operation notes were introduced in stages across the three main hospital sites: Broome (March), Derby (October), and Kununurra (November), in the Kimberley region of Australia, in 2019. The implementation consisted of the use of the Theatre Management System (TMS) software (Government of WA Department of Health, Health Applications, Enterprise Applications Network V4.7.2.4) to type operation reports and education material, both verbal and written, for the consultant general surgeons.

Quality of general surgery operation note content was evaluated in 2020 through a review of 100 operation notes (50 typed and 50 handwritten) against the RCSEng operation note guidelines 18 fields (Table 1). Two handwritten notes selected were excluded as incorrectly listed as general surgery procedures; as such, 48/50 handwritten notes were included. To the best of our knowledge, there are no equivalent Australian surgical guidelines for operation note requirements; as such, we compared content against well-established United Kingdom guidelines. Three additional fields were included: unplanned return, surgical count correct, and author breakdown (Consultant, Registrar, Junior Medical Officer, or Unclear). Cases were selected from TMS general surgery lists using a random number generator for date selection each month in 2020. Average general surgery operation numbers during the busy tourist season (April to September) are between 100-120/month, with significantly fewer operations during the wet season (October to March). Cases were then selected and reviewed electronically and by recalling medical records by a single resident medical officer who was not rostered to the general surgery department and not an author of the operation notes.

**Table 1:** Operation note quality. Operation note guideline fields from the RCSEng are situated in the left column labelled “Standard”. On review, it was noted whether the standard was “Present”, “Absent”, or “Not applicable” for both typed operation notes (T) and handwritten operation notes (H). Incision was deemed “not applicable” for endoscopic procedures, which were included as performed in the main operating theatre by a consultant general surgeon. RCSEng: The Royal College of Surgeons of England; DVT: Deep Venous Thrombosis

Standard	Present		Absent		Not Applicable	
	T	H	T	H	T	H
Date and time	50	45	0	3	0	0
Elective/emergency procedure	50	5	0	43	0	0
Patient details	50	48	0	0	0	0
Names of the operating surgeon and assistant	50	46	0	2	0	0
Name of the theatre anaesthetist	0	0	50	48	0	0
Unplanned return to theatre	50	0	0	48	0	0
Operative procedure carried out	50	45	0	3	0	0
Incision	33	23	0	13	17	12
Operative diagnosis	50	46	0	2	0	0
Operative findings	50	44	0	4	0	0
Any problems/complications	6	7	0	0	44	41
Any extra procedure performed and the reason why it was performed	8	30	0	18	42	0
Details of tissue removed, added or altered	44	45	0	3	6	0
Identification of any prosthesis used, including the serial numbers of prostheses and other implanted materials	0	2	2	5	48	41
Details of closure technique	30	30	0	5	20	13
Surgical count correct	50	20	0	28	0	0
Anticipated blood loss	1	0	49	48	0	0
Antibiotic prophylaxis (where applicable)	15	15	10	9	25	24
DVT prophylaxis (where applicable i.e.- day cases exempt)	8	4	11	0	31	44
Detailed postoperative care instructions	49	48	1	0	0	0
Abbreviations and acronyms	44	37	6	11	0	0
Signature (or electronic signature)	50	46	0	2	0	0

Staff perceptions of communication post typed operation note implementation were reviewed through an anonymous, opt-in, electronic survey across emergency departments, general practices, nursing staff, and allied health staff members from Broome, Derby, and Kununurra. The electronic survey was emailed from a central official email address (Medical Director) to all three main hospital sites and general practitioner (GP) practices. Questions were answered using a set five-point Likert scale (Appendix 1). The study was approved by the WA Country Health Service Human Research Ethics Committee (Approval: RGS0000004229).

## Results

General surgery operation note quality 

**Figure 1:**
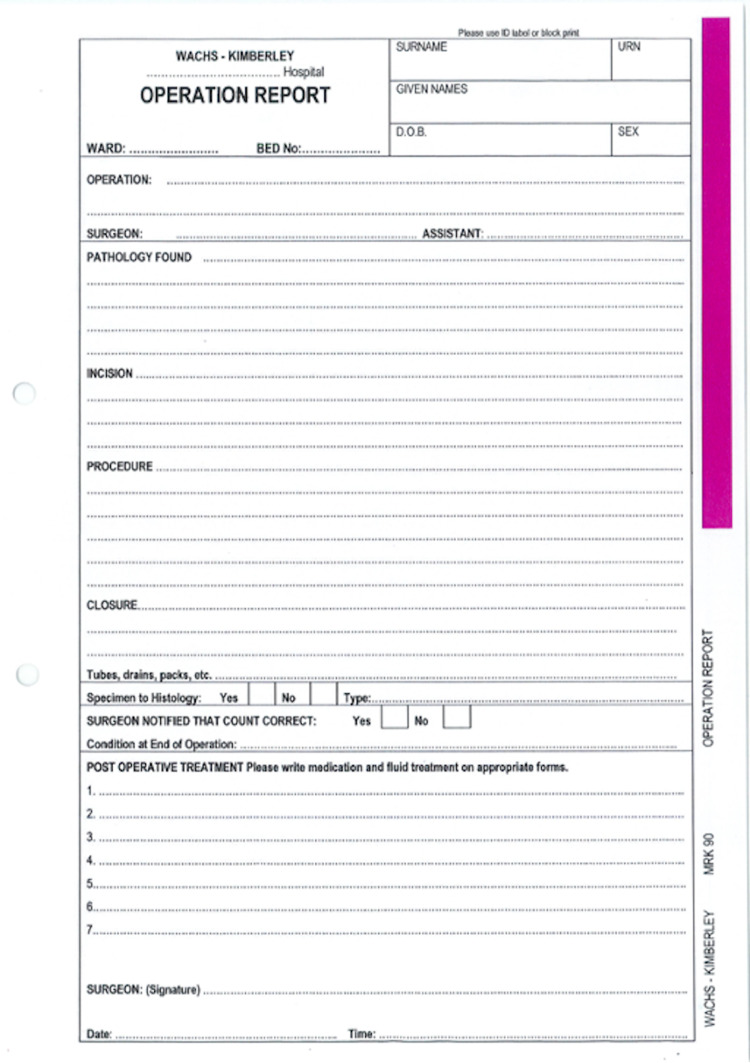
Western Australia Country Health Service operation report template.

Handwritten notes (n=48) using a standard template (Figure 1) were 100% congruent with two of the RCSEng 18 fields: patient details and post-operative plan. Typed operation notes (n=50) had a 100% congruence with 10 of the 18 fields: date, time, elective/emergency, patient details, operating surgeon and assistant, operative procedure, diagnosis, findings, and signature (Table 1). 

For typed notes there was also 100% congruence with two additional fields: unplanned return to theatre and surgical count correct, compared to zero and 42% (20/48) for handwritten notes, respectively. Both methods failed to record the anaesthetist’s name and only one (typed) recorded the estimated blood loss. Abbreviation and acronym use was high for both methods: 88% (44/50) in typed and 77% (37/48) in handwritten. 

Author breakdown showed 66% (33/50) of typed operation notes were completed by surgical registrars compared to 90% (43/48) of handwritten notes completed by consultant general surgeons. Operation notes with no abbreviation use (six typed and 11 handwritten) were by consultant authors.

General surgery operation note staff perceptions

A total of 79 responses were received from all three sites: allied health professionals (n=10), general practitioners (n=18), emergency department doctors (n=20), and nursing staff (n=31).

Of the respondents, 60% reported that handwritten notes had a negative impact on timely post-operative care (either through accessibility or legibility) compared to greater than 70% “rarely or never” for typed. All typed notes were legible compared to 58% “sometimes legible” handwritten notes and more than 80% requiring assistance from colleagues to read. Of the respondents, 55% reported post-operative instructions were “sometimes” clearly stated in handwritten notes compared to 78% “always or usually” in typed operation notes.

Although not supported by reviewed operation notes, there was a perceived improvement with acronyms and abbreviations in typed notes with 43% responding “usually” for handwritten notes compared with 26% “usually” for typed. Similarly, there was a perceived improvement of documentation completion with more than 75% “usually or always” completed for typed compared to handwritten notes which were 27% “rarely” completed.

Overall, the introduction of typed operation notes was well received with more than 80% of multidisciplinary staff wanting an extension for after-hour use and 94% indicating it would be useful for all surgical specialties.

## Discussion

In 2019, the top four wholly preventable sentinel events in Australia all involved perioperative surgical care (wrong site, wrong patient, wrong procedure, and unintended retention of foreign object). Whilst The Australian Commission on Safety and Quality in Health Care have acknowledged and addressed the Australian sentinel event list with interventions such as perioperative checklists, the number of reported sentinel events in WA is on the rise [6]. Whilst the impact of suboptimal communication on these cases is not clear, replacing handwritten operation notes with electronic versions may aid in reducing these numbers. In WA public hospitals, registrars commonly write the operation notes and whilst registrar quality has previously been reviewed [8], this study provides additional insight into rural applicability and consultant performance. 

Predictably, typed operation notes increased legibility to 100% and, thus, is the preferred method for recording operation notes [4]. In addition, pre-loaded data (date/time, patient details, etc.) and mandatory fields (surgical count correct, etc.) increased recorded information and quality of content when compared to handwritten notes. Free text fields of anticipated blood loss (one typed note) and abbreviation use (44 typed and 37 handwritten) showed user-dependent areas for improvement and may benefit from increased mandatory fields or dropdown options. The most used abbreviation was “RPAO” (Routine Post Anaesthetic Observations), which has the potential to confuse or distract from optimal post-operative care and, thus, should not be used. Interestingly, all operation notes without abbreviations (six typed and 11 handwritten) were by consultant authors. This may be reflective of clinical experience, medicolegal experience, or reduced time pressures compared to the surgical registrars. 

Although no formal training for optimal operation note content exists for registrars, in order to improve the identified free text areas of deficit, education is required. This would need to involve leadership from consultant general surgeons, ongoing auditing, and the development of systems to improve quality and compliance. Further areas for improvement include the capability of incorporating clinical drawings or images into operation reports.

The high proportion of typed operation notes (66%) by surgical registrars are likely due to aligning rostered working hours with TMS access and previous experience with the system, as TMS is also used throughout WA. Likewise, after-hours rostering is limited to the consultant general surgeon on call, with access limited to handwritten operation notes (90%). Despite rostering considerations, many consultant general surgeons in the Kimberley are on short locum contracts with variable previous exposure to WA systems. Given this, consultant preference likely also contributed to the number of handwritten notes, as the majority of operations occur within working hours.

Restricted after-hours TMS access during 2019 was limited secondary to appropriate training of theatre nursing staff. TMS is also used for local auditing (morbidity and mortality) purposes, and as such appropriate training was deemed necessary to avoid errors such as incorrect patient details or operations. This has been identified as a necessary step to increasing the availability of TMS access in the future.

Local access to typed operation notes provided accessibility (where there was a detailed post-operative plan) for allied health and enhanced communication between theatre and the ward for both nursing and medical staff. In addition to increased local accessibility, TMS is also the preferred program in metropolitan areas of WA, meaning notes can be viewed across all public hospital sites providing continuity of care in metropolitan centres. Whilst this may aid multidisciplinary hospital-based interaction, communication with GPs outside of hospital systems remains an area for improvement.

For all surgical cases, a copy of the operation note is either directly faxed to the GP or incorporated into a discharge summary. When local GPs were surveyed, handwritten notes were more than 90% “rarely or never” available within 24 hours post-operation, compared with 50% for typed operation notes. If not immediately accessible at time of discharge, the timeframe of availability for handwritten was: days (50%) or weeks (11%), compared to minutes (5%), hours (33%) or days (39%) for typed. Whilst this data shows increased accessibility, it still shows room for improvement with regard to systematic processes for our primary care practitioners.

Increased legibility and accessibility to operation notes will also improve future auditing and research capabilities, improve "lost" or incorrectly filed notes, ensure safer morbidity and mortality reviews with a mandatory field of “unplanned return to theatre” and potentially increase hospital funding through improved coding.

There are some limitations to this study. As it was a single centre rural hospital, the results may not be applicable to the rest of regional/rural WA or Australia. It was a retrospective review, and although steps were made (randomisation and external reviewer of content), this could have introduced selection bias. Although a survey of staff perceptions was reviewed, the actual adverse impact of post-operative care was not investigated or compared between the two operation note styles.

## Conclusions

The Kimberley region poses a unique set of challenges to the continuity of post-surgical care. As such, clear, effective, and prompt communication between multidisciplinary team members is paramount to patient safety. A part of this communication is through the quality of documentation and accessibility of operation notes. This review shows typed operation notes improved legibility by 100% and congruence with established guidelines by 45%. It also exhibits a successful model that is compatible with both local and metropolitan multidisciplinary access across remote WA for timely post-operative care.

Possible future suggestions as a result of this data would be to continue to use typed operation notes as the preferred method of recording intra-operative data, with the view to extend its use to after-hours and other surgical specialties. Ongoing auditing of operation note quality would help guide further training for both surgical registrars and consultants, in addition to consideration of increasing mandatory fields or dropdown options. Lastly, the evolution of Australian-based guidelines would be welcomed.

In an unprecedented time where elective surgical procedures are being reduced to meet pandemic demands, now is the time to review, consider, and institute practices that improve intra-operative and post-operative care.

## Appendices

Appendix 1: Operation Note Survey.

1. Are you currently or have you ever worked as a doctor, nurse or allied health professional in the Kimberley? 

ED/ GP/ Nurse/ Allied Health

2. Where were you/ are you currently employed? (Mark only one oval)

Broome/ Derby/ Kununurra

3. How often are *handwritten* operation notes legible?

always/ mostly/ sometimes/ rarely/ never/ not sure

4. How often are *typed* operation notes legible?

always/ mostly/ sometimes/ rarely/ never/ not sure

5. How often do you require assistance from colleagues to read *handwritten* operation notes?

always/ mostly/ sometimes/ rarely/ never/ not sure

6. How often do you require assistance from colleagues to read *typed* operation notes?

always/ mostly/ sometimes/ rarely/ never/ not sure

7. How often are the post-operative instructions clearly stated on *handwritten* operation

notes?

always/ mostly/ sometimes/ rarely/ never/ not sure

8. How often are the post-operative instructions clearly stated on *typed* operation notes?

always/ mostly/ sometimes/ rarely/ never/ not sure

9. How often are acronyms and abbreviations present on *handwritten* operation notes?

always/ mostly/ sometimes/ rarely/ never/ not sure

10. How often are acronyms and abbreviations present on *typed* operation notes?

always/ mostly/ sometimes/ rarely/ never/ not sure

11. Regarding *handwritten* operation notes, documentation is complete (all procedural information is recorded to the best of your knowledge)?

always/ mostly/ sometimes/ rarely/ never/ not sure

12. Regarding *typed* operation notes, documentation is complete (all procedural information is recorded to the best of your knowledge)?

always/ mostly/ sometimes/ rarely/ never/ not sure

13. *Handwritten* operation details are received (faxed, emailed or printed) within 24 hours of patient discharge?

always/ mostly/ sometimes/ rarely/ never/ not sure

14. *Typed* operation details are received (faxed, emailed or printed) within 24 hours of patient discharge?

always/ mostly/ sometimes/ rarely/ never/ not sure

15. Regarding *handwritten* operation notes, If not immediately accessible at time of discharge (for example- no access to iSOFT), when requesting the operation note- on average how long does this process take?

seconds/ minutes/ hours/ days/ weeks/ not sure

16. Regarding *typed* operation notes, If not immediately accessible at time of discharge (for example- no access to iSOFT), when requesting the operation note- on average how long does this process take?

seconds/ minutes/ hours/ days/ weeks/ not sure

17. Regarding *handwritten* operation notes: relevant, up-to-date information is immediately at hand and easy to locate, or searchable (physical accessibility)?

always/ mostly/ sometimes/ rarely/ never/ not sure

18. Regarding *typed* operation notes: relevant, up-to-date information is immediately at hand and easy to locate, or searchable (physical accessibility)?

always/ mostly/ sometimes/ rarely/ never/ not sure

19. Regarding *handwritten* operation notes: the information recorded correctly reflects the event being documented?

always/ mostly/ sometimes/ rarely/ never/ not sure

20. Regarding *typed* operation notes: the information recorded correctly reflects the event being documented?

always/ mostly/ sometimes/ rarely/ never/ not sure

21. How often do you use ‘iSOFT’ to review operation notes?

daily/ weekly/ monthly/ yearly/ never

22. How often have *handwritten* operation notes negatively impacted timely postoperative care? (either through accessibility, legibility etc.)

always/ mostly/ sometimes/ rarely/ never/ not sure

23. How often have *typed* operation notes negatively impacted timely postoperative care? (either through accessibility, legibility etc.)

always/ mostly/ sometimes/ rarely/ never/ not sure

24. How often have *handwritten* operation notes contributed to medication errors or missed medications (for example IV antibiotics or venous thromboembolism (VTE) prophylaxis)?

always/ mostly/ sometimes/ rarely/ never/ not sure

25. How often have *typed* operation notes contributed to medication errors or missed medications (for example IV antibiotics or VTE prophylaxis)?

always/ mostly/ sometimes/ rarely/ never/ not sure

26. How often have *handwritten* operation notes led to inappropriate diet (i.e. either prolonged fasting or wrong diet type)?

always/ mostly/ sometimes/ rarely/ never/ not sure

27. How often have *typed* operation notes led to inappropriate diet (i.e. either prolonged fasting or wrong diet type)?

always/ mostly/ sometimes/ rarely/ never/ not sure

28. How often have *handwritten* operation notes led to missed allied health referrals (unclear or missing discharge information)?

always/ mostly/ sometimes/ rarely/ never/ not sure

29. How often have *typed* operation notes led to missed allied health referrals (unclear or missing discharge information)?

always/ mostly/ sometimes/ rarely/ never/ not sure

30. Any further comments? (free text field)

## References

[1] (2022). Department of Primary Industries and Regional Development: Kimberley. http://www.drd.wa.gov.au/regions/Pages/Kimberley.aspx.

[2] (2022). Good Medical Practice: A Code of Conduct for Doctors in Australia. Good Medical Practice: A Code of Conduct for Doctors in Australia.

[3] (2022). Australian Commission on Safety and Quality in Health Care: Action 1.16 healthcare records. https://www.safetyandquality.gov.au/standards/nsqhs-standards/clinical-governance-standard/patient-safety-and-quality-systems/action-116.

[4] (2022). Royal College of Surgeons of England: 1.3 Record your work clearly, accurately and legibly. https://www.rcseng.ac.uk/standards-and-research/gsp/domain-1/1-3-record-your-work-clearly-accurately-and-legibly/.

[5] Sokol DK, Hettige S (2006). Poor handwriting remains a significant problem in medicine. J R Soc Med.

[6] (2022). The state of patient safety and quality in Australian hospitals. The State Of Patient Safety And Quality In Australian Hospitals 2019.

[7] Braaf S, Manias E, Riley R (2011). The role of documents and documentation in communication failure across the perioperative pathway. A literature review. Int J Nurs Stud.

[8] Nzenza TC, Manning T, Ngweso S, Perera M, Sengupta S, Bolton D, Lawrentschuk N (2019). Quality of handwritten surgical operative notes from surgical trainees: a noteworthy issue. ANZ J Surg.

